# Analysis of EST data of the marine protist *Oxyrrhis marina*, an emerging model for alveolate biology and evolution

**DOI:** 10.1186/1471-2164-15-122

**Published:** 2014-02-11

**Authors:** Renny Lee, Hugo Lai, Shehre Banoo Malik, Juan F Saldarriaga, Patrick J Keeling, Claudio H Slamovits

**Affiliations:** 1 Canadian Institute for Advanced Research, Program in Integrated Microbial Biodiversity, Alberta, Canada; 2 Department of Biochemistry and Molecular Biology, Dalhousie University, B3H4R2 Halifax, NS, Canada; 3 Botany Department, University of British Columbia, V6T1Z4 Vancouver, BS, Canada

**Keywords:** Dinoflagellates, Alveolates, Chromatin, Genome, Oxyrrhis

## Abstract

**Background:**

The alveolates include a large number of important lineages of protists and algae, among which are three major eukaryotic groups: ciliates, apicomplexans and dinoflagellates. Collectively alveolates are present in virtually every environment and include a vast diversity of cell shapes, molecular and cellular features and feeding modes including lifestyles such as phototrophy, phagotrophy/predation and intracellular parasitism, in addition to a variety of symbiotic associations. *Oxyrrhis marina* is a well-known model for heterotrophic protist biology, and is now emerging as a useful organism to explore the many changes that occurred during the origin and diversification of dinoflagellates by virtue of its phylogenetic position at the base of the dinoflagellate tree.

**Results:**

We have generated and analysed expressed sequence tag (EST) sequences from the alveolate *Oxyrrhis marina* in order to shed light on the evolution of a number of dinoflagellate characteristics, especially regarding the emergence of highly unusual genomic features. We found that *O. marina* harbours extensive gene redundancy, indicating high rates of gene duplication and transcription from multiple genomic loci. In addition, we observed a correlation between expression level and copy number in several genes, suggesting that copy number may contribute to determining transcript levels for some genes. Finally, we analyze the genes and predicted products of the recently discovered Dinoflagellate Viral Nuclear Protein, and several cases of horizontally acquired genes.

**Conclusion:**

The dataset presented here has proven very valuable for studying this important group of protists. Our analysis indicates that gene redundancy is a pervasive feature of dinoflagellate genomes, thus the mechanisms involved in its generation must have arisen early in the evolution of the group.

## Background

The dinoflagellate *Oxyrrhis marina* is emerging as a popular model to study many aspects of heterotrophic protist biology including ecophysiology, behaviour, distribution and dispersal, swimming, motility as well as various aspects of cellular and nuclear biology [1]. Crucially, *O. marina* is well suited to explore the origins and the unusual characteristics of two important groups of protists, dinoflagellates and apicomplexans. In this regard, *Oxyrrhis* represents an early branch within the dinoflagellate lineage. Its phylogenetic position has now been securely established as radiating close to the separation between apicomplexans and ‘crown’ dinoflagellates but after the oyster parasite *Perkinsus marinus*[2,3]. The status of *Oxyrrhis* as a dinoflagellate is not unanimous among protistologists [4,5] but the basis for including it in the group, albeit as a divergent early representative are sound [5]. Regardless the preferred taxonomic treatment, *Oxyrrhis* offers a unique perspective to understand the evolution of these fascinating protists.

Dinoflagellates are known for their highly divergent features, such as expansive genomes, an unusual karyokinetic process and a very atypical chromatin structure, unique among eukaryotes [6-10]. Apicomplexans, on the other hand, exhibit some contrasting features such as a highly developed specialization for intracellular parasitism. Both groups have unusual organellar genomes, characterized by gene loss or transfer to the nucleus and unusual genomic architecture. Compared to many heterotrophs, *O. marina* is a robust organism that is easy to maintain in the laboratory; it grows fast and has flexible nutritional requirements [11,12]. These advantages explain in part why *O. marina* is a fashionable model organism, but lack of molecular data has been a severe limitation to the scope of questions that can be addressed with this species.

Over the last few years, we have carried out several studies using a dataset of expressed sequence tags (EST) from strain CCMP1788 of *O. marina*, which was used to addressed several specific questions on plastid evolution, lateral gene transfer, the structure of the mitochondrial genome and others [3,13-15]. Specifically, this dataset has revealed that at least eight genes are likely to have been inherited from a plastid-bearing ancestor while some of them showed strong signal of being related to genes from peridinin-containing dinoflagellates and apicomplexans [13], supporting the idea that the apicoplast and the photosynthetic plastid of dinoflagellates share an origin [13,16-18]. It has also revealed well-supported examples of horizontal gene transfer (HGT) [14,15], one of which involved the acquisition of rhodopsin proteins, which may have important functional implications [14]. Finally, EST data allowed a comprehensive characterization of the mitochondrial genome of *O. marina*, providing valuable insight into the complicated scenario of the evolution of these organelles in alveolates [3,19,20]. These examples highlight the value of the data generated by the *O. marina* EST project. More recently, Lowe et al. published a transcriptomic analysis of *O. marina* isolate 44-PLY01 (Plymouth Harbour, UK) based on 454 pyrosequencing, which constitutes the first attempt to use massively parallel DNA sequencing on this species [21]. Here we report the analysis of the full EST dataset, which is now available in its entirety in public databases, and give a general overview of the nature of the genes encoded in the *O. marina* genome, with particular discussion on the evolution of the nuclear genome and chromatin architecture.

## Methods

### Strain, cultivation and EST library construction

*Oxyrrhis marina* strain CCMP 1788 was cultivated in Droop’s Ox-7 medium at the Bigelow Laboratory for Ocean Sciences (formerly CCMP). 20 L of culture was harvested in a continuous-flow centrifuge and stored in Trizol reagent (Invitrogen, Carlsbad, CA). Total RNA was prepared in 20 ml batches according to the manufacturer’s directions, resulting in 2 μg of total RNA. A directional cDNA library from polyadenylated RNA was constructed in pBluescript II SK using EcoR1 and XhoI sites (Amplicon Express, Pullman, WA, USA), and shown to contain 5.3×10^5^ cfu. 23,702 clones were picked and 5′-end sequenced using Sanger capillary sequencers (National Research Council, Halifax, NS, Canada). Quality control and vector trimming resulting in 18,012 EST sequences (deposited into GenBank EST database with accession numbers EG729650-EG747671) that assembled into 9,876 unique clusters using tbESTdb [22]. The clustering method implemented in tbESTdb is based on the phred/phrap algorithms [23] and ensures high discriminatory power to identify closely related paralogues and distinct gene copies [22]. The clusters were further examined manually using Geneious Pro versions 5 and 6 (Biomatters, Auckland, New Zealand) to assess quality. Sequences shorter than 200 bases were discarded because we observed a large proportion of low-quality and low-complexity, resulting in a filtered dataset of 8,141 sequences.

### Annotation and functional classification

Sequences in the final dataset were searched against the NCBI non-redundant (nr) protein database using BLASTN and BLASTX to identify and annotate rRNA genes and protein coding genes, respectively. BLASTX was run two times, both with the default parameters except the cut-off E-value, which was set to ≤ 1e^-10^ and ≤ 1e^-5^ for each of the two BLASTX sessions. For clusters not yielding a hit in the first search (≤ 1e^-10^), we examined their hits in the second search set (≤ 1e^-5^) individually to distinguish spurious and useful matches. BLAST searches were done using Koriblast 3.0 (Korilog SARL, Questembert, France). The top match for each sequence was kept and the taxonomic affinity recorded for each entry. High-level taxonomic assignment was done manually. Assignment of functional categories and gene ontology to the top BLASTX hits was done with Blast2go [24]. Various sequence analyses and manipulations involving sequence alignments, conceptual translation, protein sequence examinations and calculations (e.g. molecular weight, isoelectric point) were carried out with Geneious v5.6 and v6. In addition, clusters with no hits to known proteins were searched for Pfam domains with Blast2Go (Additional file 1: Table S1).

### Identification of meiotic components

Conserved proteins identified in lists of DNA repair and recombination proteins from the genome projects of *Homo sapiens*[25,26], *Saccharomyces cerevisiae* (http://db.yeastgenome.org), *Trypanosoma brucei* and *T. cruzi*[27,28], *Trichomonas vaginalis*[29] and refs. [30-33] were used to search a local database of inferred proteins from the genome sequences of *Cryptosporidium parvum*, *Toxoplasma gondii* (the smallest and largest sequenced apicomplexan genomes, respectively) and the oyster parasite *Perkinsus marinus* by batch BLASTP with an e-value cutoff of 1e^-1^. Similarly, *C. parvum*, *H. sapiens* and *S. cerevisiae* protein homologs were used to query the apicomplexan *Ascogregarina taiwanensis* genome survey sequence [34] by batch tBLASTn with an e-value cutoff of 1e^-1^. *C. parvum, T. gondii, P. marinus, H. sapiens* or *S. cerevisiae* inferred proteins were used to search the O. marina ESTs by tBLASTn with an e-value cutoff of 1e^-1^, and the identity of the best sequence hit(s) in *O. marina* verified by BLASTx against GenBank’s non-redundant database. Phylogenetic analyses of individual candidate proteins were conducted with PhyML (http://www.atgc-montpellier.fr/phyml/) [35] using the amino acid conceptual translations aligned with MAFFT [36]. The maximum likelihood trees were built using the LG substitution model with invariant sites and 8 γ-distributed substitution rate categories. Node support was assessed with 1,000 bootstrapping replicates.

### Real-time PCR estimation of relative expression

RNA from *O. marina* cultures was extracted and purified with Aurum Total RNA mini kit (Bio-Rad, Hercules, CA) and cDNA was produced using Superscript III reverse transcriptase (Invitrogen, Carlsbad, CA). cDNA was quantified with a Qubit 2.0 fluorometer (Life Technologies, Carlsbad, CA). Sets of primers were designed for *O. marina* TVP1, Actin, Tubulin and Proteorhodopsin genes using the program PrimerSelect (DNASTAR, Inc., Madison, WI, USA) with its default parameters for real-time PCR. Tests for promiscuous binding were done using Blastn. Primers were ordered from Integrated DNA Technologies Inc., (Coralville, IA, USA). A full list of primer sequences and characteristics can be found in Additional file 1: Table S2. Real-time PCR was performed with a CFX96 instrument (Bio-Rad) and iQ SYBR Green Supermix (Bio-Rad). Expression level was expressed in relative units by entering the Ct value into the standard curves prepared for each gene as described [37].

## Results and discussion

### ESTs and assembled clusters

Although now superseded by ultra-high throughput sequencing methods, cloning based cDNA library construction followed by directional Sanger sequencing remains a powerful way to analyse the gene complement of an organism because high quality, long read sequence offers the possibility to obtain full-length sequences of individual clones. We used this method to conduct a genome-wide survey of expressed genes in *O. marina*, aiming to shed light on some aspects of alveolate biology and evolution.

A total of 23,702 clones were sequenced from the 5’ end, of which 18,012 remained after quality filtering and vector trimming. The ESTs were assembled using the tbESTdb pipeline [22], resulting in 9,876 unique clusters (unigenes, [38]). Visual inspection of the clusters revealed that a large number of the short clusters were low complexity repeats, thus we decided to discard all sequences shorter than 200 bases in order to prioritize the quality of the data, resulting in a final set of 8,141 clusters. The size distribution of ESTs is bimodal, with a peak between 650 and 750 bases and another between 50 and 150 bases (not shown), suggesting that substantial of degradation in the RNA sample took place.

### Taxonomic distribution of blast hits

We used NCBI Blast to assign putative functional identity by similarity with the aid of Koriblast and Blast2go. Searching with the Blastn algorithm against NCBI’s non-redundant nucleotide database (nr) identified 6 clusters matching rRNA genes: four clusters correspond to pieces of the fragmented mitochondrial rRNA genes, which have already been analysed [3], one to nuclear small subunit RNA (SSU), and two to the nuclear large subunit RNA gene (LSU) (Table 1). Both SSU and LSU transcripts are very abundant compared to most RNA species in the sample, with 88 and 172 ESTs, respectively.

**Table 1:** **Identity of the *O. marina *** **EST clusters encoding mitochondrial transcripts and nuclear ribosomal RNA genes**

**Gene**	**Clusters**	**Total ESTs**
cox1	1	339
cob-cox3 fusion	1	130
LSU-E	1	14
LSU-E	1	53
LSU-rna10	1	18
LSU-G	1	1
Nuclear SSU	1	88
Nuclear LSU	2	172

For each type, the number of distinct clusters and the number of ESTs per clusters is indicated.

**Figure 1:**
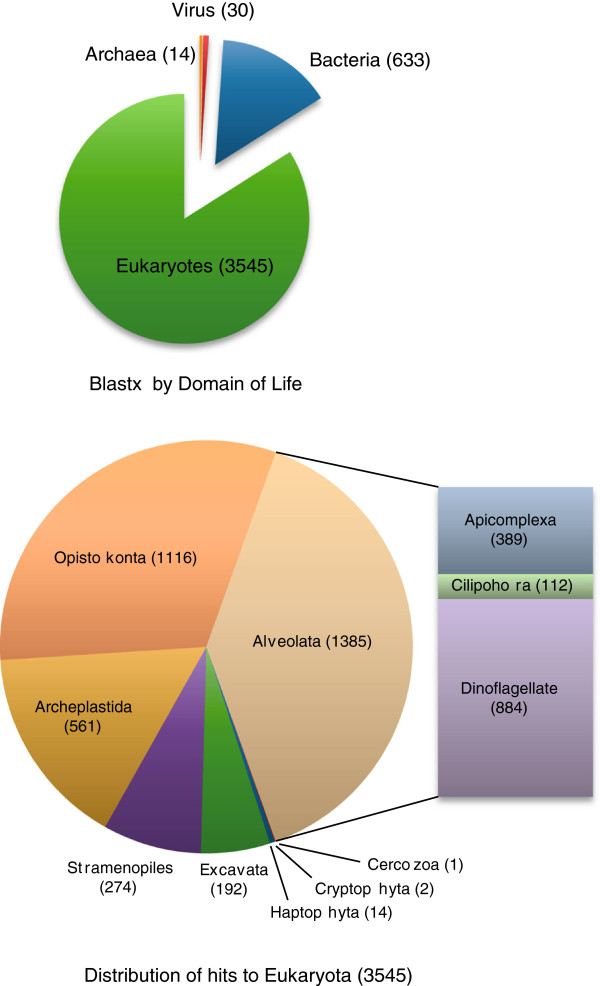
**Taxonomic distribution of the top blastx hits of 4,222 EST clusters (this set excludes clusters with no hits or spurious hits at the ≤ 1e-5 level).** Top: Number of hits by domain of life. Bottom: Distribution of eukaryotic top hits by high-order taxonomy.

Having excluded rRNA genes and sequences shorter than 200 bases, we conducted Blastx searches against the nr database with a cutoff E value set to e ≤ 1x-05. The search produced 4,515 sequences with matches. Examining alignments with lower Blast scores, we noticed that many were false positive results due to spurious similarity between repeats in the clusters and low-complexity protein sequences in the database, so we set a stricter cutoff at E ≤ 1x-10, resulting in 4,222 hits. Of these, 633 corresponded to bacteria, 30 to viruses and 14 were similar to members of Archaea (Figure 1). The remaining 3,545 positive hits were similar to eukaryotic sequences. Figure 1 shows the distribution of the eukaryotic hits by taxonomic groups: alveolates make up about one third of the total (1,385 clusters), then opisthokonts (metazoan and fungi) with 1,116 and Archaeplastida (plants, green and red algae) with 561. The remaining fraction was composed of Stramenopiles (e.g. diatoms, brown algae, oomycetes), excavates (e.g. euglenids, kinetoplastids, parabasalia), haptophytes, cryptophytes and cercozoans. Within alveolates, most top hits were from dinoflagellates (884) followed by apicomplexans (389) and ciliates (112). Not surprisingly, the largest part of the dinoflagellate hits comes from a single species, the oyster parasite *Perkinsus marinus* with (the genus *Perkinsus* is often classified in its own Phylum Perkinsozoa [4,39]). *P. marinus* is the only member of the dinoflagellate lineage with a genome project at an advanced stage (at the time of writing a presumably complete set of genes have been annotated and deposited in Genbank, but the analysis has not yet been published). (Note added during revision: a draft genome project of *Symbiodinium sp*. was published recently [40]). Dinoflagellates are still comparatively underrepresented in databases, especially in the protein databases as most sequences produced so far have been deposited as ESTs. Hits to apicomplexan species are not as conspicuous as one would expect considering that Apicomplexa is the sister taxon to dinoflagellates and there are about ten complete genomes from apicomplexan parasites in public databases. This could be a consequence of the high degree of specialization that characterizes the phylum Apicomplexa, typically exhibiting high divergence of protein sequences and heavy gene loss. Sequences with top Blast similarity to animals and fungi collectively (i.e. Opisthokonta) represent a similar fraction as alveolates (Figure 1). This set of genes probably represents a core of well-conserved and ubiquitous eukaryotic genes of very deep ancestry, a class of genes that usually exhibits little correlation between Blast similarity and phylogenetic affinity (and also likely reflects the large number of animal and fungal genomes that are available). The remaining one-third of Blast hits correspond to plants, green and red algae, stramenopiles and excavates (Figure 1). In part, these assignments are probably due to similar factors as those from animals and fungi, but could also include sequences with different evolutionary histories for at least two reasons. First, all alveolates, or at least the clade conformed by apicomplexans and dinoflagellates [17] descend from plastid-harbouring ancestors and as such, their nuclei contain many genes derived from that ancient photosynthetic endosymbiont that may be contributing to the hits to archaeplastids and stramenopiles. Eight genes likely to be inherited from a plastid from this sample have been reported in a previous paper [13]. On the other hand, however, if these genes were really derived from the endosymbiont, they might be expected to be found in ciliate genomes as well, but the evidence for this is still controversial [41-43]. Second, *O. marina* is a voracious predator and its diverse menu includes mainly green and red algae as well as many stramenopiles and haptophytes. Continuous repeated exposure to prey could have resulted in a number of genes being transferred and integrated into the nuclear genome [44].

**Figure 2:**
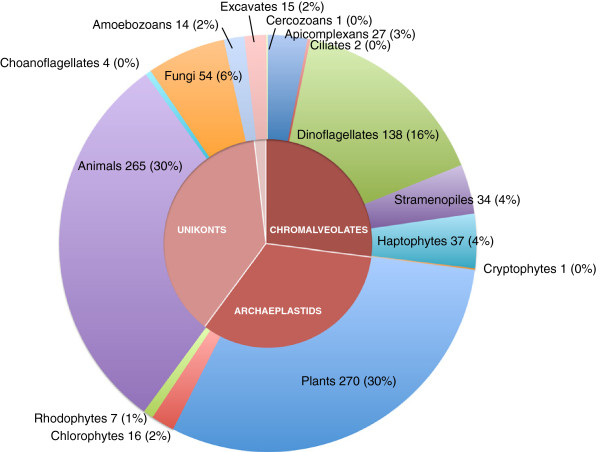
**EST clusters of *O. marina *with no hits to NCBI’s non-redundant protein database were searched against the NCBI’s ‘est-others’ database using tblastx (compares translated amino acid sequences using nucleotide queries and database).** The pie chart shows the taxonomic distribution of the top hits (≤ 1e-5). Number of hits and percentage are shown. The central circle indicates major eukaryotic groupings of the portions in the external circle.

Overall, roughly half of the sequences had no significant matches to known proteins deposited in the nr database. Clearly, the fraction of sequences with no Blastx matches in GenPep depends on the abundance of sequences from related organisms in the database and on how atypical is the organism in its gene content and degree of sequence divergence. As more genomes in a group of related organisms are sequenced and annotated, the first factor becomes less relevant and the fraction of unmatched sequences decreases. Figure 2 shows the distribution of top Blast hits when we searched NCBI’s “est-others” database using the *O. marina* ESTs that returned no significant hits in the previous search. The preponderance of plants and animals simply reflects the largely biased composition of the database, whereas the next fraction in abundance corresponds to dinoflagellates in spite of the comparatively low representation of these protists in the database. The fraction of unmatched Blast sequences in *O. marina* at the E ≤ 1xe-10 threshold is 47%, which is in line with other estimates: 53% for *Alexandrium catenella*[45], 71% for *Karenia brevis*[46], 63% for a combined set from *Amoebophrya sp*. and *Karlodinium veneficum*[47] and 72% for *A. minutum*[48]. Variation is due to a mix of factors, including non-standardized criteria to determine cutoff, potential biases from library construction and sample size and increasingly better representation of dinoflagellate genes in the databases. We also compared our EST dataset to the transcriptomic data generated by 454 pyrosequencing by Lowe et al. [21]. Even though the number of clusters (contigs) in both studies is roughly similar (ca. 8,000), the overlap in sequence identity was very low. Only 23% of our EST dataset (sequences 200 bp or longer) had one or more blastn hits (E ≤ 1xe-5) among the 7,398 sequences in the 454 dataset. While the two samples come from closely related organisms, we also used tblastx to compare at the predicted amino acid level in case divergence at the nucleotide level was too high. This time the overlap increased slightly to 29%.

### Bacterial sequences

**Figure 3:**
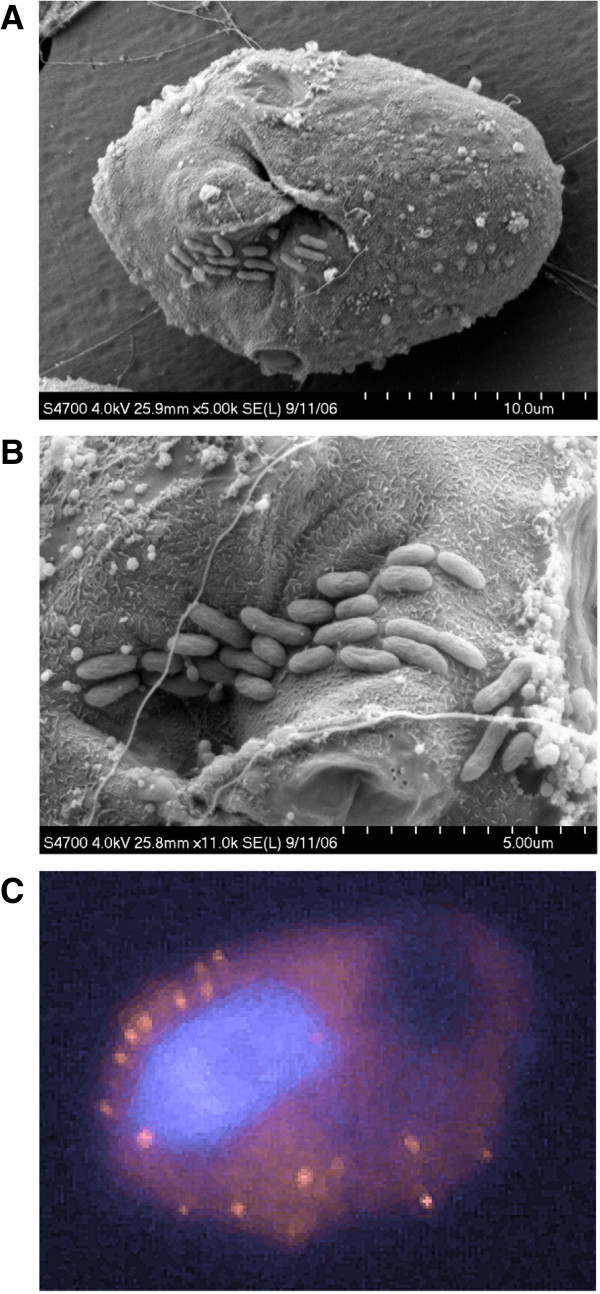
***Oxyrrhis marina *cells coexist with bacteria, probably in some type of symbiotic relationship.** The figure shows *O. marina* cells from antibiotic-treated cultures in close relationships with unidentified bacteria. **A, B**: Scanning electron micrographs showing the oral region of *O. marina* with rod-shaped bacteria attached. **C**: Fluorescence in-situ hybridization of an *O. marina* cell using a fluorescent probe that recognizes a conserved region of bacterial small subunit (16S) ribosomal RNA genes following the standard protocol described in http://www.arb-silva.de/fish-probes/fish-protocols/. Blue: DAPI-stained nucleus of *O. marina*; red dots: bacteria.

The ESTs from *O. marina* contains 633 sequences for which the top Blast hit is bacterial. Many of those probably reflect sampling artifacts that result in misleading top Blast hits and others might represent genes with an evolutionary origin different from the *O. marina* nucleus (i.e. horizontal and endosymbiotic gene transfer). We also detected a sizeable fraction with very high similarity to alpha-proteobacteria of the order Rhodobacterales, mainly from the genus *Oceanicaulis*. We examined these sequences closely and they do not appear to be derived from genuine, polyadenylated mRNA. First, they are extremely similar, often identical to the annotated genome of the bacterium *O. alexandrii*. Second, the orientation of the coding sequence appears to be equally distributed between forward and reverse, and third, some individual ESTs even contain portions of genes that are adjacent in the *O. alexandrii* genome. We conclude that these sequences are most likely derived from a contamination with bacterial DNA during the library construction. *O. alexandrii* has been isolated from cultures of the dinoflagellate *Alexandrium* and several Rhodobacterales are known to coexist with dinoflagellates, even as symbiotic partners [49-53]. Likely, symbiotic bacteria that resist methods to generate axenic cultures accompany the *O. marina* culture. We observed that *O. marina* cells that had been grown with antibiotics still contain tightly associated bacteria (Figure 3). Overall, the *O. alexandrii* Blast matches accounted for 82 of the genes with top hits to bacteria (13%, not shown), leaving a sizable number of genes, many of which could still come from symbionts but others may have resulted from HGT. Unfortunately there are no available data to confirm this possibility, since our ESTs typically do not include the spliced leader sequence characteristic of the 5′ end of dinoflagellate genomes [46,54].

### Extensive gene redundancy

In many cases, ESTs apparently encoding the same gene were assembled as separate clusters of highly similar sequence (albeit below the strict threshold for assembly). Close examination of the raw files revealed that this is not due to sequencing errors or low quality, but to the presence of genuinely distinct copies of many genes, in some cases over 40. Multi-copy genes have been described previously in dinoflagellates, often as tandems of as many as 5,000 adjacent copies [55,56] and recently, hints for disperse arrangements affecting many genes have been reported by sequencing [21,57] and fluorescent in situ hybridization [58] techniques. The pyrosequencing-based transcriptomic study by Lowe et al. detected a number of redundant genes, including tandem arrangements [21], but we did not find extensive overlap in multicopy genes between both studies: only three genes (hsp70, hsp90 and S-adenosyl-methionine synthetase) in our list from Table 2 were also found to be redundant in the Lowe et al. analysis.

**Table 2:** **Identity inferred as top BlastX hits of the *O. marina *** **genes with largest numbers of distinct clusters**

**Hit accession**	**Clusters**	**Total Ests**	**Definition**	**Species**
AAO14677	42	240	Proteorhodopsin	*Pyrocystis lunula*
ABV72550	25	87	DVNP	*Heterocapsa triquetra*
ZP_07751542	23	152	GPR1/FUN34/yaaH family protein	*Mucilaginibacter paludis*
BAE79387	13	41	Actin	*Symbiodinium sp. CS-156*
ABV22332	12	132	Cysteine protease 1	*Noctiluca scintillans*
XP_002506839	12	61	Acetate-coa ligase	*Micromonas sp. RCC299*
XP_002500277	11	27	Acetyltransferase-like/FAD linked oxidase	*Micromonas sp. RCC299*
AAM02973	10	90	Heat shock protein 70	*Crypthecodinium cohnii*
XP_002775922	10	27	Succinate dehydrogenase, putative	*Perkinsus marinus*
XP_002780969	10	25	Heterogeneous nuclear ribonucleoprotein, putative	*Perkinsus marinus*
ACI12882	9	122	NAD-dependent alcohol dehydrogenase	*Euglena gracilis*
CBJ30560	8	20	Glutathione S-transferase	*Ectocarpus siliculosus*
XP_002786250	8	8	40S ribosomal protein S9, putative	*Perkinsus marinus*
AAW79379	7	36	Fumarate reductase	*Heterocapsa triquetra*
XP_002787701	7	35	14-3-3 protein, putative	*Perkinsus marinus*
ZP_06799564	7	8	Hypothetical protein	*Mycobacterium tuberculosis*
ABI14419	6	39	Heat shock protein 90	*Karlodinium micrum*
AAV71134	6	23	Cytosolic class II fructose bisphosphate aldolase	*Heterocapsa triquetra*
ABF22754	6	16	Mitochondrial cytochrome c oxidase subunit 2b	*Karlodinium micrum*
ACA60905	6	15	Gag-pol polyprotein	*Thalassiosira pseudonana*
ZP_07985860	6	12	ATP-dependent DNA helicase	*Streptomyces sp.*
YP_002500649	6	6	Peptidase C14, caspase catalytic subunit p20	*Methylobacterium nodulans*
XP_002765341	5	46	S-adenosylmethionine synthetase, putative	*Perkinsus marinus*
ABG56231	5	38	Translation elongation factor-like protein	*Karlodinium micrum*
XP_001763482	5	23	Acetyl-CoA synthetase	*Physcomitrella patens*
XP_002184734	5	22	Predicted protein	*Phaeodactylum tricornutum*
XP_002769616	5	21	Conserved hypothetical protein	*Perkinsus marinus*
NP_001068397	5	17	Hypothetical protein	*Oryza sativa*
AAG01128	5	17	Hypothetical protein	*Solanum lycopersicum*

Accumulating evidence suggests that prevalence of multicopy genes in dinoflagellates is more common than in other organisms [57,59-62], but a thorough assessment of the prevalence of gene redundancy in one organism is lacking. In our sample, among the sequences with positive Blast hits, 422 were represented by two or more distinct clusters, 64 sequences by 4 or more clusters and 11 genes were represented by 10 to 42 clusters (Table 2). Since ESTs are clustered only if they are highly similar, different clusters with the same blast hits probably represent different genomic loci, but the opposite may or may not be true: genes present in multiple but identical copies cannot be recognized by this approach, since all ESTs originating from all the units will cluster in a single contig. Tandem arrangements of identical copies have been described in dinoflagellates, therefore they may occur in *O. marina* as well. In fact, Lowe et al. found evidence of tandemly arranged genes encoding Beta and Alpha tubulin, EF-2, Rhodopsin and HSP90 with intergenic spacers ranging between 200 and 400 bp [21].

In contrast to the protein-coding genes, we found that expressed nuclear rRNA genes are highly homogeneous: all 88 ESTs from small subunit cluster to a single contig, whereas the 172 ESTs from the large subunit cluster to 2 contigs (Table 1). Long operons of identical or nearly identical units of rRNA genes are very common in eukaryotic genomes, and the high level of similarity among the copies has been attributed to gene conversion and other mechanisms that result in concerted evolution [63]. The sequence heterogeneity observed in many protein-coding genes may reflect particular genomic conditions that prevent them from achieving or maintaining homogeneity. However, concerted evolution depends on genomic and biological factors such as the spatial distribution of the repeated genes (i.e. whether tandemly arranged or scattered) and the frequency of somatic and meiotic recombination, all features almost virtually unknown in dinoflagellates.

### Levels of gene expression and gene variants

We observed large variations in the number of ESTs per cluster (Tables 2 and 3). As reported previously, the most abundant corresponded to mitochondrial coxI, while mitochondrial cob-coxIII fusion was also among the top with 130 ESTs ([3], Table 1). Of the nucleus-encoded genes, proteorhodopsin was the most highly expressed ([14]). Aside for housekeeping genes typically highly expressed in eukaryotic cells (hsp90, actin, tubulins, EFL, ribosomal proteins), the list of most abundant ESTs is populated almost exclusively by metabolic proteins, suggesting that at the time of harvesting the cells were in active metabolic state (Additional file 2: Figure S1). Notable among the metabolic genes are alcohol dehydrogenase and the glyoxylate cycle enzyme isocitrate lyase, suggesting active utilization of 2C molecules such as ethanol or acetate as carbon sources. Interestingly, another highly expressed gene is Gpr1/Fun34/YaaH, whose product is essential for acetate permease activity in *Aspergillus* and its mutation trigger hypersensitivity to acetic acid in yeast [64,65]. When grown with prey food, *O. marina* cells behave voraciously and one individual can easily ingest three or four prey cells, which in the case of the green alga *Dunaliella tertiolecta*, are about half the size of an *O. marina* cell. Consistently, we observed evidence of intense protein degradation activity in the form of high expression levels of cysteine protease 1 (132 ESTs, Table 3). Three enzymes of the S-adenosyl-L-homocysteine metabolism are also highly expressed: adensyl homocysteine hydrolase, adenosyl methionine synthetase and adenosyl homocysteinase (Table 3), which participate in several metabolic pathways, mainly the synthesis of adenosine, methionine and cysteine.

**Table 3:** **Identity inferred as top BlastX hits of the *O. marina *** **genes with largest numbers of ESTs**

**Hit accession**	**Clusters**	**Ests**	**Definition**	**Species**
AAO14677	42	240	Proteorhodopsin	*Pyrocystis lunula*
ZP_07751542	23	152	GPR1/FUN34/yaaH family protein	*Mucilaginibacter paludis*
ABV22332	12	132	Cysteine protease 1	*Noctiluca scintillans*
ACI12882	9	122	NAD-dependent alcohol dehydrogenase	*Euglena gracilis*
AAM02973	10	90	Heat shock protein 70	*Crypthecodinium cohnii*
ABV72550	25	87	DVNP	*Heterocapsa triquetra*
ZP_01726360	2	63	Aldehyde dehydrogenase	*Cyanothece sp.*
XP_002950429	3	62	S-Adenosyl homocysteine hydrolase	*Volvox carteri*
XP_002506839	12	61	Acetate-coa ligase	*Micromonas sp.*
XP_002765341	5	46	S-adenosylmethionine synthetase, putative	*Perkinsus marinus*
XP_002784353	3	43	H + -translocating inorganic pyrophosphatase TVP1,	*Perkinsus marinus*
YP_130418	3	42	L-lactate permease	*Photobacterium profundum*
BAE79387	13	41	Actin	*Symbiodinium sp.*
ABI14419	6	39	Heat shock protein 90	*Karlodinium micrum*
ABG56231	5	38	Translation elongation factor-like protein	*Karlodinium micrum*
AAW79379	7	36	Fumarate reductase	*Heterocapsa triquetra*
XP_002787701	7	35	14-3-3 protein, putative	*Perkinsus marinus*
XP_002766754	2	30	40S ribosomal protein S11, putative	*Perkinsus marinus*
XP_002500277	11	27	Acetyltransferase-like/FAD linked oxidase	*Micromonas sp. RCC299*
XP_002775922	10	27	Succinate dehydrogenase, putative	*Perkinsus marinus*
ABD46571	4	27	Alcohol dehydrogenase-like protein	*Euglena gracilis*
XP_002786429	4	27	Osmotic growth protein, putative / Fumarate reductase	*Perkinsus marinus*
XP_002904993	2	26	Isocitrate lyase	*Phytophthora infestans*
XP_002780969	10	25	Heterogeneous nuclear ribonucleoprotein, putative	*Perkinsus marinus*
ZP_06800645	3	25	Heat shock protein	*Mycobacterium tuberculosis*
XP_002773236	1	25	Ribonucleotide reductase small subunit, putative	*Perkinsus marinus*
ABV22229	3	24	ATP/ADP translocator	*Karlodinium micrum*
YP_638223	3	24	Nucleotide-diphosphate-sugar epimerase/NmrA family protein	*Mycobacterium sp. MCS*
AAV71134	6	23	Cytosolic class II fructose bisphosphate aldolase	*Heterocapsa triquetra*
XP_001763482	5	23	Acetyl-CoA synthetase	*Physcomitrella patens*
XP_001638515	3	23	No hits	
CBX99834	2	23	Similar to cytochrome b2	*Leptosphaeria maculans*
XP_002786953	2	23	Tubulin alpha chain, putative	*Perkinsus marinus*
XP_002184734	5	22	Predicted protein	*Phaeodactylum tricornutum*
ABF61766	3	22	Chloroplast 3-dehydroquinate synthase/O-methyltransferase	*Heterocapsa triquetra*
XP_002788505	3	22	Hypothetical protein	*Perkinsus marinus*
ABU52986	2	22	Beta-tubulin	*Karenia brevis*
XP_002911883	9	21	Hypothetical protein	*Coprinopsis cinerea*
XP_002769616	5	21	Conserved hypothetical protein	*Perkinsus marinus*
XP_002780466	3	21	2-methylcitrate synthase, putative	*Perkinsus marinus*
CBJ30560	8	20	Glutathione S-transferase	*Ectocarpus siliculosus*
XP_666127	3	20	Ribosomal protein L5A	*Cryptosporidium hominis*
AAN31463	1	20	Glutamine synthetase	*Phytophthora infestans*
ACV41934	4	19	No hits	
ACJ13434	3	19	Adenosylhomocysteinase	*Amphidinium carterae*
XP_002766763	2	19	Protein TIS11, putative	*Perkinsus marinus*
XP_001612035	2	18	Conserved hypothetical protein	*Babesia bovis*
NP_001068397	5	17	Hypothetical protein	*Oryza sativa*
AAG01128	5	17	Hypothetical protein	*Solanum lycopersicum*
AAX27763	4	17	Hypothetical protein	*Toxoplasma gondii*
ABI13175	2	17	Asparaginyl endopeptidase	*Emiliania huxleyi*
XP_002776404	1	17	Methylenetetrahydrofolate reductase, putative	*Perkinsus marinus*
ABI14188	1	17	ADP-ribosylation factor	*Pfiesteria piscicida*
ABF22754	6	16	Mitochondrial cytochrome c oxidase subunit 2b	*Karlodinium micrum*
XP_002765511	2	16	40S ribosomal protein S3a, putative	*Perkinsus marinus*
XP_002772672	2	16	Vacuolar ATP synthase subunit b, putative	*Perkinsus marinus*

At face value, these numbers suggest that some genes are relatively highly expressed; however to test whether this has any correspondence to mRNA levels in the cell, we conducted real-time quantitative PCR (qPCR) on a sample of genes. Specifically, mRNA levels of four genes, proteorhodopsin (PR), TVP1, alpha tubulin (Atub) and actin were quantified from *O. marina* culture grown in similar conditions as the original culture. In coincidence with the estimation from EST abundance, PR and TVP1 were first and second in the qPCR estimation with 50,000 and 18,100 relative units, respectively (not shown). For Atub and actin 4,200 and 1,000 relative units were estimated, respectively. Albeit preliminarily, this test shows correspondence between the number of ESTs for a given gene and the qPCR estimation of gene expression, but conclusions based on this evidence must be taken with caution until a more rigorous experiment is conducted. There are many factors, both biological and technical that could explain differences between EST abundance and qPCR estimation [21,66]. In this case, the multicopy structure of the genome may constitute an additional complication since qPCR primers pick only a restricted sample of the mRNAs encoding a particular type of protein, resulting in potentially large sampling errors.

Moreover, it is now emerging that in dinoflagellates gene expression is largely modulated posttranscriptionally [61,62,67-69]. If so, transcriptional regulation may have little relationship to the protein levels, and increasing the number of functional transcriptional units could be an alternative way to maintain high levels of mRNA of certain genes. In our data, highly expressed genes tend to exhibit more distinct variants (Tables 2 and 3), raising the intriguing possibility that dinoflagellates modulate baseline expression levels by, at least in part, increasing the number of copies of the gene instead of (or in addition to) adjusting transcription levels.

### Functional categorization of the *O. marina* ESTs

#### DNA repair and meiosis

We identified 27 *O. marina* transcripts homologous to genes conserved in humans, yeast, and other protists that were functionally linked to the recognition and repair of damaged DNA in model animals or fungi [25,26] (Additional file 1: Table S3). These include components of the excision repair machinery, DNA double-strand break repair by homologous recombination (HR), editing and processing nucleases (EPN), post-replication repair (PRR), chromatin structure (CS), the DNA damage checkpoint (DDC), DNA replication licensing (DRL), and DNA damage response (DDR). Excision repair protein homologs encoded by *O. marina* include (i) break excision repair (BER) poly (ADP-ribose) polymerase PARP2 that protects single-strand DNA interruptions, (ii) mismatch repair (MMR) protein Mlh1, a mutL homolog also required for meiotic crossovers, and for (iii) nucleotide excision repair (NER), replication factor A (RFA1) that binds to sites of DNA damage, and the XPD/ERCC2 5′-3′ helicase that helps unwind the pre-incision intermediate. Homologs of DNA polymerase catalytic subunits delta, epsilon and PCNA, employed in MMR and NER, were also identified in *O. marina*. Components of the HR machinery include the SbcD 3′ exonuclease homolog Mre11, RecA recombinase homolog Rad51, Brca1, a sister chromatid cohesin subunit (Smc3), homologous condensin subunits Smc2 and Smc4, and meiosis-specific Hop2 and Spo11-2 (Additional file 2: Figure S1 and Additional file 3: Figure S2). Conserved homologs of a flap endonuclease (FEN1), the DNA damage response and checkpoint signaling machinery (Suc1, Rad17, Chk1, Chk2) and the DNA replication licensing complex (Mcm3, Mcm5 and Mcm7) are also encoded by *O. marina*. Components of the post-replication repair Rad6 pathway (Rad6A, Rad6B) are present. Proteins involved in chromatin structure such as BLM, RecQ helicases are also identified.

These findings indicate that *O. marina* encodes conserved components of several eukaryotic recombination and repair pathways, except for non-homologous end joining (NHEJ). Since the proteins encoded by these genes interact together with other conserved DNA repair proteins where studied in other eukaryotes, we expect that additional *O. marina* genomic or transcriptomic data will reveal homologous genes encoding other key DNA repair and recombination proteins, including additional members of the ERCC, XRCC and Rad52 epistasis groups, additional MutL and MutS homologs involved in mismatch repair, and more meiosis-specific homologs.

To date, a single report on sexual reproduction has been published for *O. marina*[70,71]. Based on observations of small cells presumed to be gametes, the paper claims that *O. marina* cells engage in sexual reproduction, but no data support the occurrence of meiotic division [72]. Moreover, even the ploidy status and most details about the life cycle of *O. marina* are poorly known [58,72]. Since *O. marina* encodes meiosis-specific Spo11-2 and Hop2 genes, we expect other “core meiotic genes” [73,74] not yet detected might also be present. Since Spo11-2 and Mre11 genes are present, we expect to find Rad50, since Rad50 and Mre11 act together in other eukaryotes to remove Spo11 from DNA ends in meiosis and also process DNA ends during mitotic HR. Since Hop2 is present, meiosis-specific Mnd1 and Dmc1 homologs might also be encoded, since in other eukaryotes Hop2 and Mnd1 form a complex that interacts with Dmc1 in interhomolog strand exchange. Presence of these pieces of the conserved meiotic machinery indicates that meiosis is indeed part of the life cycle of *O. marina*, although probably in a very inconspicuous way. Possibly the conditions in which we generated the RNA (i.e. exponential growth) favours asexual reproduction, hence the paucity of meiosis-related genes in our sample. This also means that the life cycle must include diploid (or polyploidy) stages.

#### Chromatin architecture and remodeling

**Figure 4:**
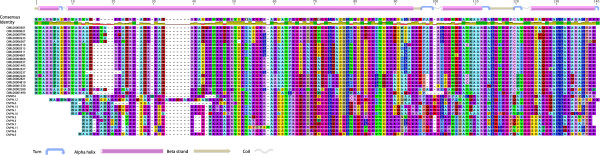
**Graphic alignment of twenty full-length variants of the nuclear protein DVNP from *O. marina *(OML) with 13 DVNP sequences from *Hematodinium sp*.** The alignment shows strong sequence and structural conservation, although not at the level observed in typical eukaryotic histones. A schematic representation of the predicted structural features based on the consensus sequence is shown at the top. The pattern of a long alpha helix followed by a ‘helix-turn-helix’ terminal motif is generally conserved among all the variants.

Dinoflagellates have long been known as ‘rule breakers’ because they present exceptions to many well-established rules of eukaryotic cell biology. For example, numerous lines of evidence suggest that typical nucleosomal organisation of the chromatin is absent in dinoflagellates, and what histones remain do not function in the same capacity as in other eukaryotes [8,9]. Instead, the chromatin appears to be rich in basic proteins but the way in which nuclear DNA and proteins interact is still unknown [6,7,10,75,76]. This raises the fundamental question of the involvement of chromatin organisation for transcriptional regulation in dinoflagellates. Gene regulation through chromatin remodeling is a ubiquitous eukaryotic feature that exhibits variations but the essential aspects are presumed to be present in all eukaryotes. Evidence that histone genes are present and indeed expressed in dinoflagellates is starting to emerge, suggesting that these proteins probably play some role in chromatin organisation [75,77]. Since chromatin organisation at the molecular level appears to be typical in *Perkinsus*, the most basal lineage of the dinoflagellate tree for which genomic data is available, the data from *O. marina* could provide valuable hints on the early stages of the transformations leading to the unusual nature of the dinoflagellate chromatin. We looked for evidence of histones and chromatin remodeling sequences in *O. marina* and found no clear histone homologues; neither the typical eukaryotic nor the histone-like proteins of bacterial origin that have been reported in *Crypthecodinium*[78]. We did find one sequence with high similarity (E < 1x10^-48^) to a histone deacetylase of the AcuC/AphA family and another with similarity to Sir2, another conserved histone deacetylase of the Sirtuin family involved in epigenetic silencing. Even if we assume that histones should be present as suggested by recent findings on other species, failure to find transcripts in our sample is not surprising given that their evidence has remained elusive in several other studies and when found, histone transcripts binned among the lowly expressed genes. In animals and plants, replication-dependent histone transcripts are not polyadenylated, and in yeasts, the length of the polyA tail of histones varies with the stage of the cell cycle. Difficulty in detecting histone transcripts in dinoflagellates may also reflect the existence of similar mechanisms of transcriptional regulation involving short or absence of polyA tails. A recent study in the parasitic dinoflagellate *Hematodinium sp.* described a novel protein named DVNP (for Dinoflagellate Viral Nuclear Protein), which appears to be a main basic protein found in the chromatin [75]. In the study, micrococcal nuclease digestion of intact *Hematodinium* chromatin failed to yield the typical nucleosomal band pattern on an agarose gel, unlike *P. marinus*, which yield the 180 bp ladder expected from partial nucleosomal DNA digestion [75]. The concomitant presence of these two features in *Hematodinium* and not in *Perkinsus* suggests that the loss of nucleosomal organization of the nuclear DNA is somehow related to the replacement of histones by DVNP as the main basic nuclear protein [75]. Interestingly, sequences with high similarity to DVNP were also found among ESTs of different dinoflagellates, including *O. marina*. On this information, we searched exhaustively our data and found 25 clusters with high similarity to DVNP (Tables 2 and 3). Of these, we analysed the amino acid translations of the 20 sequences that encompassed the complete protein (Figure 4). The proteins ranged between 134 and 142 amino acids in length and had a mean isoelectric point of 12.73, indicating a strong basic character. The predicted mean molecular weight was 14.8 KDa. The proteins exhibit secondary structure features similar to those found by Gornik et al. [75] in the *Hematodinium* DVNP: an alpha helix of variable length encompassing the first half of the protein followed by a ‘helix-turn-helix’ region (Figure 4). The *O. marina* DVNP sequences are predicted to have nuclear localization signals (NLS), a feature also found in the *Hematodinium* proteins [75].

As DVNP appear to be well established in *O. marina*, they must have taken their present role prior to the split between *O. marina* and the core dinoflagellates, but after the split of *P. marinus*[75]. Very likely, DVNP is the true identity of Np23, the major basic nuclear protein detected previously in nuclear extracts of *O. marina* cells [79]. Notwithstanding, the presence of histone deacetylase genes suggests that histones and other associated factors are still functional in dinoflagellates, therefore it cannot be ruled out that at least part of the genome is arranged with the canonical nucleosomal organisation. Clearly more comprehensive genomic sequencing and molecular biology experiments must be done in order to determine what other conserved elements of chromatin and epigenetic regulation are involved in these protists.

#### Transcription and RNA processing

While the amount of dinoflagellate sequence data is increasing, the components of gene regulation in the group have largely been left unexplored, despite the recent claims that much of the regulation of dinoflagellate genes is controlled at the post-transcriptional level [67,68,80,81]. However, documenting the presence of the canonical (or better-studied) regulatory pathways is also needed to determine the complexity of gene regulation. For instance, mRNA splicing and transcription are two broad categories that may be valuable to dissect in dinoflagellates. Splicing is of particular interest because there is extremely little information of introns and their features in the group, despite its large genome sizes. Further, the idea that all dinoflagellate mRNAs are trans-spliced with a leader sequence [46,54] adds an additional layer of complexity to splicing and gene regulation.

We searched the *O. marina* clusters for the major mRNA splicing and transcription components using a consolidated list of 256 splicing proteins and 228 transcription proteins. A component was considered present if the *O. marina* cluster also wasn’t identified via blasting from a different conserved splicing or transcriptional component. Additional file 1: Table S4 lists the genes involved in splicing or transcription with matches in our *O. marina* dataset, and their functions. In addition, potential genes of interest in this category contain Pfam domains for RNA recognition and/or binding and various types of DNA-binding domains such as zinc fingers and knuckles (Additional file 3: Figure S2). From the subset of splicing genes identified, the majority of them are either associated with the U6 or U2 snRNPs – the spliceosome parts that recognize the intron in the initial steps on splicing [82]. These include the Sm and Sm-like (Lsm) proteins. Curiously, the U6 snRNP is also the major spliceosome component that is known to participate in leader trans-splicing [83], so over-representation of U6 snRNP components may suggest elevated expression due to involvement in trans-splicing. Prp46 and Cwc2, also identified, are members of the Nineteen Complex (NTC) that acts in the first major step of splicing. Not identified in this EST survey includes the most conserved splicing protein (Prp8), although its particular constituent domains were (data not shown). Length limitations of the ESTs may also have prohibited the clear identification of the major transcription factors and the RNA polymerases involved in transcription – only auxiliary transcriptional components were identified, with many of those participating in processes unrelated to mRNA transcription.

#### Retroelements

Evidence for active retroelements that could provide reverse transcriptase (RT) activity could help us understand some of the unusual characteristics of dinoflagellate genomes [84,85]. In particular, endogenous RT would be necessary for a hypothesis that dinoflagellate mRNAs are frequently retrotranscribed into dsDNA and integrated into the genome [84]. This process would result in the creation of large numbers of retrogenes that accumulate in the genome, and may partially explain the large numbers of highly expressed genes. This model is supported by the presence of ‘relic’ spliced leaders (rSL) immediately after the SL sequence that caps every mRNA at the 5′ end. The rSL appear in a sizeable fraction of mRNAs from several species [60,84] and are thought to be remnants of previous events of processing and recycling. The most common sources of endogenous RT activity in eukaryotic cells are LTR and non-LTR retrotransposons and telomerases, therefore we searched our EST data for sequences with similarity to known RT proteins but also to other features found in LTR and non-LTR transposons. Table 4 shows 14 clusters that were found to have similarity (E value < 1.10^-10^) to retrotransposons, all matching different regions of LTR-transposons belonging to a single class, known as Ty1/copia. In addition, clusters unidentifiable by Blastx but containing Pfam domains related to TN functions are listed in the Additional file 1: Table S1. Ty1/copia is one of the two main types of LTR-retrotransposons, is ubiquitous in eukaryotes and has been most widely studied in plan genomes. LTR-transposons are capable of mobilization via a ‘copy-and-paste’ mechanism involving transcription of the element and making DNA copies by an RT protein encoded by the transcript itself. The replicative activity of retrotransposons is hindered most of the time to avoid the deleterious effects of their proliferation. This is achieved epigenetically by methylation of certain regions of the LTR that otherwise act as promoters, but under certain conditions, transcription is unleashed allowing the elements to replicate and proliferate. Uncontrolled bursts of retrotransposon activity can result in occupying large proportions of genomes in short periods of time, events that and are thought to have played and a vital role in the organisation and evolution of eukaryotic genomes. Outside these episodic and apparently rare events of proliferation, transcripts of retrotransposons occur at very low levels, if at all. Since the level of expression of retrotransposons may be a strong predictor of active transposition, we wondered if the transcripts we found in our data are indication that transposition is ongoing in *O. marina*. Unfortunately, it is very difficult to make meaningful comparisons with expression data from other organisms in absolute terms because every transposon-genome system is different and there are no comparative analysis done. Instead, we can compare the expression of the *O. marina* elements (as revealed by the number of ESTs) relative to other genes. Ty1/copia clusters in *O. marina* are expressed at low to moderate levels (1 to 20 ESTs per cluster), but collectively they add up to 97 ESTs (Table 4). If the number of ESTs representing a gene in the sample even as a rough approximation of its relative level of expression, Ty1/copia element is among the top in transcript abundance (compare to Table 3) and it would be reasonable to speculate that RT proteins are present. This is an interesting possibility because it would lend support to the hypothesized role of mRNA recycling in dinoflagellate genome evolution by showing that retrotransposons can be a suitable source of RT activity [84,85].

**Table 4:** ***O. marina *** **EST clusters with top Blastx hits corresponding to known transposable elements**

**Cluster ID**	**Top hit Acc.**	**Top hit**	**E-value**	**ESTs**
OML00000073	XP_002422173	Hypothetical protein FOXB_16913 Fusarium oxysporum	8.00E-27	7
OML00000330	ABA95820	Hypothetical protein FOXB_16913 Fusarium oxysporum	3.00E-38	20
OML00002280	ACB59199	Copia-like protein [Brassica oleracea]	7.00E-15	3
OML00002762	EFY94000	Retrotransposon like protein [Metarhizium anisopliae ARSEF 23]	8.00E-12	3
OML00002917	ABF93649	Retrotransposon protein Ty1-copia subclass [Oryza sativa Japonica group]	5.00E-18	9
OML00002925	CAB46043	Retrotransposon like protein [Arabidopsis thaliana]	4.00E-16	5
OML00004005	BAB01972	Copia-like retrotransposable element [Arabidopsis thaliana]	2.00E-10	12
OML00004886	AAD32898	Putative retroelement pol polyprotein [Arabidopsis thaliana]	1.00E-16	1
OML00005041	AAP46257	Putative polyprotein [Oryza sativa Japonica Group]	8.00E-15	4
OML00006583	BAB01972	Copia-like retrotransposable element [Arabidopsis thaliana]	6.00E-12	8
OML00009617	XP_003376336	Retrovirus-related Pol polyprotein from transposon TNT 1-94 [Trichinella spiralis]	2.00E-13	11
OML00010486	EGU73258	Hypothetical protein FOXB_16913 Fusarium oxysporum	2.00E-21	6
OML00010490	BAB01972	Copia-like retrotransposable element [Arabidopsis thaliana]	3.00E-12	5
OML00010499	ABA95820	Retrotransposon protein, putative, unclassified [Oryza sativa Japonica Group]	1.00E-21	3
**Total**				**97**

### Lateral gene transfer

**Figure 5:**
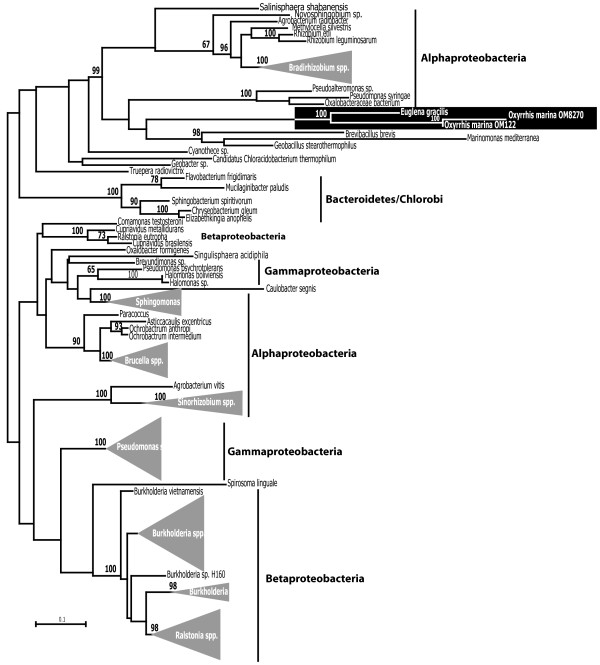
**Schematic phylogenetic tree of amino acid sequences of Zinc-dependent Alcohol dehydrogenase proteins including representatives from the main bacterial lineages and *E. gracilis *and *O. marina *(highlighted in a black box), which are the only eukaryotic organisms for which homologs have been detected.** The numbers at the nodes indicate bootstrap support when higher than 50%.

Lateral gene transfer (LGT) has already been reported from *O. marina*, including the acquisition of bacterial AroB [15] and proteorhodopsin genes at least two times independently [14]. Detection of LGT in eukaryotic sequence data is not always as clear-cut, however, since confounding factors like sequence divergence, incomplete taxon sampling and a convoluted evolutionary history, or the presence of contaminating bacteria in the culture can complicate the interpretation of the phylogenies of suspected LGT cases. When we looked for potential LGT cases in our data we encountered combinations of all these factors, in particular the presence of about 600 bacterial sequences, presumably originating from contaminant DNA. While most of these sequences can be readily identified as contaminants, they undermine the level of certainty of potential LGT. In spite of this we have identified two additional genes with a conflicting phylogenetic signal suggesting horizontal acquisition from bacteria. Both cases share an intriguing pattern of being closely associated to an unrelated eukaryote but beyond that, embedded among bacteria (similar to a growing number of other cases of LGT, see [86]). Additional file 4: Figure S3 shows a phylogenetic analysis of the deduced protein sequence of cluster OML00001921 along with 2 sequences from diatoms, one from the ichthyosporean *Sphaeroforma arctica* and 46 eubacterial sequences of L-lactate permease (LctP). The *O. marina* sequence forms a strongly supported node with the other three eukaryotic sequences (100% bootstrap), which in turn is connected to various proteobacteria, mainly involving the subgroups gamma and delta. The LctP protein catalyses the transport of L-lactate across membranes. It has been suited functionally only in a few species of bacteria, most notably in *E. coli*, where this gene is part of an operon involved in L-lactate utilisation [87]. In eukaryotes, members of the monocarboxylate transporter family (MCT) catalyze the proton-linked transport of monocarboxylates such as L-lactate, pyruvate, and the ketone bodies across the plasma membrane. Since LctP does not seem to have any relationship with the MCT family, and no other eukaryotic organisms were found to contain LctP-related sequences, an ancient, common origin of the four eukaryotic sequences shown in Additional file 4: Figure S3 is unlikely. At the same time, the fact that the eukaryotic sequences branch together to the exclusion of everything else makes it intriguing. If the lctP genes were acquired independently by *O. marina*, the diatoms and the ichthyosporean *S. arctica*, they have been transferred from the same or very similar donors. Alternatively, the gene may have been transferred from bacteria to one eukaryotic lineage, and then transferred between eukaryotes [86]. The second case is shown in Figure 5. Several clusters were found to be highly similar to alcohol dehydrogenase proteins (ADH) that seem to be absent from other eukaryotes except for one species, *Euglena gracilis*. Alcohol dehydrogenases belong in a very large superfamily of ancient origin known as MDR (medium-chain dehydrogenase/reductase) and is formed by zinc-dependent ADHs, quinone reductases, and many more families and subfamilies [88]. The most recent comprehensive study of MDR sequences identified over 500 families that can be ascribed to MDR superfamily, 8 of which are highly widespread with ADH being the largest [88]. In addition, the study recognized other 9 families of restricted scope but of special interest for their functions or potential relevance. The ADH sequences from *O. marina* are most similar to one family from this group of additional “special interest” MDR families tentatively named BurkDH family because of its prevalence among *Burkholderia* species and several other genera of proteobacteria including *Pseudomonas, Brucella, Ralstonia* and *Rhizobium* (Figure 5). Surprisingly, only one eukaryotic protein sequence can be found in Genbank that belongs in this family but it is from *E. gracilis*, a phototrophic freshwater protist completely unrelated to *O. marina*. Unfortunately we are unable to make inferences as to the adaptive roles of this acquisition by *O. marina* and *E. gracilis* as no complete genome data are available for neither organism, therefore we do not know if other MDR paralogs are present. In addition, the function of BurkDH proteins has not yet been investigated [88]. These two cases illustrate puzzling scenarios that result of great interest for understanding the evolutionary dynamics of metabolic adaptation but are in turn difficult to interpret in the context of the current data. Clearly these cases will have to be reanalyzed when more, comprehensive from free-living protists (in these cases, dinoflagellates, euglenids, haptophytes) are available.

## Conclusions

Our EST dataset from *O. marina* has so far yielded interesting insights into the evolution, genetics, phylogeny and metabolism of this species and dinoflagellates at large. Here we tapped on this valuable dataset to conduct additional investigations, this time concentrating on genes and molecular characteristics associated to nuclear and genomic biology, which is an area where dinoflagellates are particularly unusual. We describe several gene categories and show that *O. marina* contains many of the typically widespread components that comprise DNA repair, and gene expression, suggesting that in spite of the seemingly highly divergent nature of dinoflagellate nuclear processes, they still maintain many of the core eukaryotic mechanisms. Moreover, we find extensive gene redundancy and multiplicity, indicating transcription from multiple genomic loci. For some of the most highly represented transcripts, we estimate multiple genomic copies suggesting a positive correlation between transcript abundance and genomic copy number, which may be a generalized dinoflagellate feature. Extending on previous findings, we described two striking examples of lateral gene transfer, reinforcing the idea that acquisition of foreign genes plays an important role, in shaping the *O. marina* genome and further supporting the role of this phenomenon in adaptation on eukaryotes, particularly heterotrophic protists.

## Competing interests

The authors declare that they have no competing interests.

## Authors’ contributions

RL analysed data and drafted the paper, SBM conducted analysis of meiosis genes and lateral gene transfer and drafted sections of the paper, HL conducted real-time PCR experiments, JFS contributed to the data generation and analyses, PJK provided the cultures and data generation and drafted the paper, CHS conducted analyses, provided supervision and wrote the paper. All authors read and approved the final manuscript.

## Supplementary Material

Additional file 1: Table S1 PFAM domains found among the *O. marina* EST clusters with no hits to known proteins. **Table S2**- Primer sequences, melting temperature Tm (^o^C), and insert length (bp) for four *O. marina* genes. **Table S3** (next page): *O. marina* encodes DNA repair and recombination proteins conserved in other eukaryotes. Homologs of components of the machinery for base excision repair (BER), mismatch repair (MMR), nucleotide excision repair (NER), homologous recombination (HR), meiosis-specific homologous recombination (HR-M1), DNA polymerase subunits involved in repair (DNAP), editing and processing nucleases (EPN), post-replication repair (PRR), chromatin structure relevant to repair (CS), the DNA damage checkpoint (DDC), DNA replication licencing (DRL), and DNA damage response (DDR) are present in *O. marina.* Data identified in the complete genome sequences of humans (*H. sapiens*), yeast (*S. cerevisiae*), kinetoplastids (*T. brucei*), parabasalids (*T. vaginalis*), apicomplexans (*T. gondii, C. parvum,* and genome sequence survey of *A. taiwanensis*), and a dinoflagellate (*P. marinus*) is compared with the *O. marina* ESTs. **Table S4**: The *O. marina* EST dataset contains a number of sequences with hits to proteins involved in transcriptional regulation and splicing. Listed below are eciprocal hits with a database built with curated proteins from *H. sapiens* and *S. cerevisiae*. Click here for file

Additional file 2: Figure S1 *O. marina* encodes orthologs of meiosis-specific recombination genes. Aligned amino acid sites were analyzed by PhyML with an invarying and 8 γ-distributed substitution rate categories and the LG substitution model. Numbers at the nodes indicate % bootstrap support (≥ 50%) from 1000 replicates. *O. marina* Spo11 is closely related to apicomplexan Spo11-2. 218 sites, LnL = –10981.3. Click here for file

Additional file 3: Figure S2 *O. marina* encodes orthologs of meiosis-specific recombination genes. Aligned amino acid sites were analyzed by PhyML with an invarying and 8 γ-distributed substitution rate categories and the LG substitution model. Numbers at the nodes indicate% bootstrap support (≥ 50%) from 1000 replicates. *O. marina* Hop2 is most closely related to its ortholog in *Perkinsus marinus,* within the alveolates. 172 sites, LnL = –6417.0. Click here for file

Additional file 4: Figure S3 Phylogeny of representative L-lactate permease LctP proteins indicates that *O. marina lctP* is most closely related to *lctP* in diatoms and an icthyosporean (*S. arctica*)*,* which are derived from a clade of marine bacterial *lctP* homologs. 502 amino acid sites were analyzed by PhyML with an invarying and 8 γ-distributed substitution rate categories and the LG substitution model. Numbers at the nodes indicate % support (≥ 50%) from 1000 bootstrap replicates. LnL = – 23076.2. No other eukaryotic homologs were identified by BLASTp searches of the JGI, Broad Institute, or NCBI non-redundant databases, nor by tBLASTn searches of dbEST-others, with an e-value cutoff of 1. Some of the highly similar *Neisseria* and *Haemophilus* orthologous protein sequences were excluded from the phylogeny shown here. Click here for file
