# Supplementary material for: Analysis of EST data of the marine protist Oxyrrhis marina, an emerging model for alveolate biology and evolution
Source: BMC Genomics. 2014 Feb 11;15:122. doi: 10.1186/1471-2164-15-122 (PMC3942190; doi:10.1186/1471-2164-15-122)
Supplement: Additional file 1: Table S1 — PFAM domains found among the O. marina EST clusters with no hits to known proteins. Table S2- Primer sequences, melting temperature Tm (oC), and insert length (bp) for four O. marina genes. Table S3 (next page): O. marina encodes DNA repair and recombination proteins conserved in other eukaryotes. Homologs of components of the machinery for base excision repair (BER), mismatch repair (MMR), nucleotide excision repair (NER), homologous recombination (HR), meiosis-specific homologous recombination (HR-M1), DNA polymerase subunits involved in repair (DNAP), editing and processing nucleases (EPN), post-replication repair (PRR), chromatin structure relevant to repair (CS), the DNA damage checkpoint (DDC), DNA replication licencing (DRL), and DNA damage response (DDR) are present in O. marina. Data identified in the complete genome sequences of humans (H. sapiens), yeast (S. cerevisiae), kinetoplastids (T. brucei), parabasalids (T. vaginalis), apicomplexans (T. gondii, C. parvum, and genome sequence survey of A. taiwanensis), and a dinoflagellate (P. marinus) is compared with the O. marina ESTs. Table S4: The O. marina EST dataset contains a number of sequences with hits to proteins involved in transcriptional regulation and splicing. Listed below are eciprocal hits with a database built with curated proteins from H. sapiens and S. cerevisiae. [file 1471-2164-15-122-S1.docx]

Additional file 1: Table S1: PFAM domains found among the *O. marina* EST clusters with no hits to known proteins.

| **hmm acc** | **hmm name** | **type** | **hmm start** | **hmm end** | **hmm length** | **bit score** | **E-value** | **clan** | **Description** |  |
| --- | --- | --- | --- | --- | --- | --- | --- | --- | --- | --- |
| PF12796.2 | Ank_2 | Family | 1 | 56 | 89 | 35.3 | 9.4e-09 | CL0465 | Ankyrin repeats (3 copies) |  |
| PF12796.2 | Ank_2 | Family | 3 | 87 | 89 | 42.7 | 4.5e-11 | CL0465 | Ankyrin repeats (3 copies) |  |
| PF12796.2 | Ank_2 | Family | 30 | 88 | 89 | 33.7 | 2.9e-08 | CL0465 | Ankyrin repeats (3 copies) |  |
| PF12796.2 | Ank_2 | Family | 8 | 83 | 89 | 40.4 | 2.5e-10 | CL0465 | Ankyrin repeats (3 copies) |  |
| PF12796.2 | Ank_2 | Family | 22 | 81 | 89 | 37.4 | 2.1e-09 | CL0465 | Ankyrin repeats (3 copies) |  |
| PF13637.1 | Ank_4 | Domain | 6 | 53 | 54 | 30.1 | 4.3e-07 | CL0465 | Ankyrin repeats (many copies) | |
| PF13857.1 | Ank_5 | Domain | 2 | 55 | 56 | 33.5 | 2.9e-08 | CL0465 | Ankyrin repeats (many copies) | |
| PF13650.1 | Asp_protease_2 | Domain | 9 | 58 | 90 | 26.3 | 7.1e-06 | CL0129 | Aspartyl protease |  |
| PF13229.1 | Beta_helix | Family | 74 | 141 | 158 | 23.4 | 3.7e-05 | CL0268 | Right handed beta helix region | |
| PF13229.1 | Beta_helix | Family | 11 | 153 | 158 | 44.6 | 1.1e-11 | CL0268 | Right handed beta helix region | |
| PF07731.9 | Cu-oxidase_2 | Domain | 23 | 78 | 138 | 34.7 | 9.4e-09 | CL0026 | Multicopper oxidase |  |
| PF03175.8 | DNA_pol_B_2 | Family | 65 | 186 | 462 | 47.7 | 7.8e-13 | CL0194 | DNA polymerase type B, organellar and viral | DNA polymerase possibly viral |
| PF13499.1 | EF_hand_5 | Domain | 4 | 63 | 66 | 29.8 | 4.3e-07 | CL0220 | EF-hand domain pair |  |
| PF13499.1 | EF_hand_5 | Domain | 9 | 64 | 66 | 31.0 | 1.9e-07 | CL0220 | EF-hand domain pair |  |
| PF13499.1 | EF_hand_5 | Domain | 3 | 66 | 66 | 72.7 | 1.8e-20 | CL0220 | EF-hand domain pair |  |
| PF13499.1 | EF_hand_5 | Domain | 2 | 36 | 66 | 28.4 | 1.2e-06 | CL0220 | EF-hand domain pair |  |
| PF13833.1 | EF_hand_6 | Domain | 10 | 49 | 54 | 26.4 | 3.4e-06 | CL0220 | EF-hand domain pair |  |
| PF13833.1 | EF_hand_6 | Domain | 15 | 53 | 54 | 40.2 | 1.6e-10 | CL0220 | EF-hand domain pair |  |
| PF05091.7 | eIF-3_zeta | Family | 2 | 133 | 516 | 25.1 | 5.7e-06 | No_clan | Eukaryotic translation initiation factor 3 subunit 7 (eIF-3) | |
| PF04116.8 | FA_hydroxylase | Family | 7 | 57 | 114 | 31.4 | 1.8e-07 | No_clan | Fatty acid hydroxylase superfamily | |
| PF04632.7 | FUSC | Family | 49 | 246 | 650 | 36.9 | 1.4e-09 | CL0307 | Fusaric acid resistance protein family | |
| PF03398.9 | Ist1 | Family | 3 | 161 | 165 | 44.9 | 7e-12 | No_clan | Regulator of Vps4 activity in the MVB pathway | |
| PF08212.7 | Lipocalin_2 | Domain | 5 | 140 | 143 | 25.2 | 9.8e-06 | CL0116 | Lipocalin-like domain |  |
| PF05050.7 | Methyltransf_21 | Family | 1 | 83 | 166 | 24.0 | 2.8e-05 | CL0063 | Methyltransferase FkbM domain | |
| PF13847.1 | Methyltransf_31 | Domain | 2 | 84 | 152 | 27.3 | 2e-06 | CL0063 | Methyltransferase domain |  |
| PF06813.8 | Nodulin-like | Family | 81 | 214 | 248 | 27.4 | 1.6e-06 | CL0015 | Nodulin-like |  |
| PF05028.9 | PARG_cat | Family | 12 | 110 | 340 | 33.8 | 1.8e-08 | No_clan | Poly (ADP-ribose) glycohydrolase (PARG) | |
| PF08016.7 | PKD_channel | Family | 4 | 87 | 426 | 22.5 | 2.9e-05 | CL0030 | Polycystin cation channel |  |
| PF14099.1 | Polysacc_lyase | Family | 144 | 208 | 223 | 47.7 | 1.3e-12 | No_clan | Polysaccharide lyase |  |
| PF13877.1 | RPAP3_C | Domain | 5 | 93 | 94 | 55.5 | 3.5e-15 | No_clan | Potential Monad-binding region of RPAP3 | |
| PF13893.1 | RRM_5 | Domain | 4 | 56 | 56 | 27.9 | 1.4e-06 | CL0221 | RNA recognition motif. (a.k.a. RRM, RBD, or RNP domain) | RNA binding |
| PF14259.1 | RRM_6 | Domain | 1 | 60 | 69 | 27.9 | 1.5e-06 | CL0221 | RNA recognition motif (a.k.a. RRM, RBD, or RNP domain) | RNA binding |
| PF07727.9 | RVT_2 | Family | 3 | 95 | 246 | 24.8 | 8.2e-06 | CL0027 | Reverse transcriptase (RNA-dependent DNA polymerase) | Retroelement |
| PF07727.9 | RVT_2 | Family | 144 | 229 | 246 | 22.4 | 4.3e-05 | CL0027 | Reverse transcriptase (RNA-dependent DNA polymerase) | Retroelement |
| PF07004.7 | SHIPPO-rpt | Repeat | 1 | 33 | 37 | 11.3 | 0.47 | No_clan | Sperm-tail PG-rich repeat |  |
| PF13469.1 | Sulfotransfer_3 | Family | 2 | 70 | 212 | 31.0 | 3.6e-07 | CL0023 | Sulfotransferase family |  |
| PF13414.1 | TPR_11 | Repeat | 13 | 67 | 69 | 34.2 | 1.2e-08 | CL0020 | TPR repeat |  |
| PF00583.19 | Acetyltransf_1 | Family | 3 | 82 | 83 | 30.8 | 2e-07 | CL0257 | Acetyltransferase (GNAT) family | Histone acetyltransferase |
| PF00023.25 | Ank | Repeat | 2 | 31 | 33 | 30.9 | 1.3e-07 | CL0465 | Ankyrin repeat |  |
| PF01036.13 | Bac_rhodopsin | Domain | 1 | 215 | 222 | 123.8 | 5.6e-36 | CL0192 | Bacteriorhodopsin-like protein | |
| PF01036.13 | Bac_rhodopsin | Domain | 4 | 201 | 222 | 85.1 | 3.7e-24 | CL0192 | Bacteriorhodopsin-like protein | |
| PF01062.16 | Bestrophin | Family | 19 | 133 | 296 | 27.9 | 9.6e-07 | No_clan | Bestrophin, RFP-TM, chloride channel | |
| PF01062.16 | Bestrophin | Family | 42 | 174 | 296 | 31.6 | 6.8e-08 | No_clan | Bestrophin, RFP-TM, chloride channel | |
| PF01062.16 | Bestrophin | Family | 193 | 284 | 296 | 35.2 | 5.7e-09 | No_clan | Bestrophin, RFP-TM, chloride channel | |
| PF01062.16 | Bestrophin | Family | 21 | 182 | 296 | 31.9 | 5.5e-08 | No_clan | Bestrophin, RFP-TM, chloride channel | |
| PF01062.16 | Bestrophin | Family | 35 | 180 | 296 | 38.7 | 4.8e-10 | No_clan | Bestrophin, RFP-TM, chloride channel | |
| PF01062.16 | Bestrophin | Family | 218 | 291 | 296 | 28.9 | 4.7e-07 | No_clan | Bestrophin, RFP-TM, chloride channel | |
| PF01062.16 | Bestrophin | Family | 171 | 294 | 296 | 35.5 | 4.4e-09 | No_clan | Bestrophin, RFP-TM, chloride channel | |
| PF01062.16 | Bestrophin | Family | 30 | 205 | 296 | 33.4 | 2e-08 | No_clan | Bestrophin, RFP-TM, chloride channel | |
| PF00168.25 | C2 | Domain | 2 | 60 | 85 | 31.9 | 7.6e-08 | CL0154 | C2 domain |  |
| PF00168.25 | C2 | Domain | 4 | 84 | 85 | 45.0 | 6.5e-12 | CL0154 | C2 domain |  |
| PF00168.25 | C2 | Domain | 1 | 71 | 85 | 21.5 | 0.00013 | CL0154 | C2 domain |  |
| PF03381.10 | CDC50 | Family | 89 | 142 | 278 | 20.3 | 0.00022 | No_clan | LEM3 (ligand-effect modulator 3) family / CDC50 family | |
| PF00027.24 | cNMP_binding | Domain | 4 | 90 | 91 | 22.6 | 6.1e-05 | No_clan | Cyclic nucleotide-binding domain | |
| PF00027.24 | cNMP_binding | Domain | 4 | 86 | 91 | 33.1 | 3.3e-08 | No_clan | Cyclic nucleotide-binding domain | |
| PF00027.24 | cNMP_binding | Domain | 4 | 82 | 91 | 30.8 | 1.7e-07 | No_clan | Cyclic nucleotide-binding domain | |
| PF00027.24 | cNMP_binding | Domain | 3 | 32 | 91 | 24.4 | 1.6e-05 | No_clan | Cyclic nucleotide-binding domain | |
| PF00116.15 | COX2 | Domain | 21 | 120 | 120 | 125.1 | 1.1e-36 | CL0026 | Cytochrome C oxidase subunit II, periplasmic domain | |
| PF00313.17 | CSD | Domain | 2 | 65 | 66 | 25.5 | 7.2e-06 | CL0021 | 'Cold-shock' DNA-binding domain | DNA binding |
| PF00313.17 | CSD | Domain | 25 | 65 | 66 | 29.1 | 5.4e-07 | CL0021 | 'Cold-shock' DNA-binding domain | DNA binding |
| PF00034.16 | Cytochrom_C | Domain | 2 | 90 | 91 | 31.3 | 2.5e-07 | CL0318 | Cytochrome c |  |
| PF01266.19 | DAO | Domain | 254 | 342 | 358 | 33.3 | 2.2e-08 | CL0063 | FAD dependent oxidoreductase | |
| PF00035.20 | dsrm | Domain | 2 | 65 | 67 | 25.3 | 1.5e-05 | CL0196 | Double-stranded RNA binding motif | binds dsRNA |
| PF00122.15 | E1-E2_ATPase | Family | 36 | 73 | 230 | 30.0 | 2.2e-07 | No_clan | E1-E2_ATPase |  |
| PF00378.15 | ECH | Family | 3 | 144 | 245 | 131.0 | 3.3e-38 | CL0127 | Enoyl-CoA hydratase/isomerase family | |
| PF00120.19 | Gln-synt_C | Domain | 2 | 117 | 259 | 79.8 | 1.5e-22 | CL0286 | Glutamine synthetase, catalytic domain | |
| PF03951.14 | Gln-synt_N | Domain | 4 | 84 | 84 | 63.9 | 6.5e-18 | No_clan | Glutamine synthetase, beta-Grasp domain | |
| PF00300.17 | His_Phos_1 | Domain | 26 | 157 | 158 | 31.3 | 1.6e-07 | CL0071 | Histidine phosphatase superfamily (branch 1) | |
| PF01419.12 | Jacalin | Domain | 54 | 127 | 130 | 24.1 | 2.3e-05 | No_clan | Jacalin-like lectin domain |  |
| PF02373.17 | JmjC | Family | 68 | 107 | 114 | 22.6 | 8.8e-05 | CL0029 | JmjC domain, hydroxylase | histone modification; heterochromatinization; nucleoside modification |
| PF00013.24 | KH_1 | Domain | 15 | 58 | 58 | 35.2 | 6.2e-09 | CL0007 | KH domain | binds RNA or ssDNA |
| PF01764.20 | Lipase_3 | Family | 1 | 133 | 142 | 85.2 | 2.5e-24 | CL0028 | Lipase (class 3) |  |
| PF00112.18 | Peptidase_C1 | Domain | 125 | 188 | 220 | 26.0 | 6.6e-06 | CL0125 | Papain family cysteine protease | |
| PF00112.18 | Peptidase_C1 | Domain | 125 | 207 | 220 | 58.7 | 6.4e-16 | CL0125 | Papain family cysteine protease | |
| PF04749.12 | PLAC8 | Family | 2 | 102 | 106 | 34.1 | 2.9e-08 | No_clan | PLAC8 family |  |
| PF00636.21 | Ribonuclease_3 | Family | 4 | 52 | 114 | 30.2 | 4.5e-07 | No_clan | Ribonuclease III domain | binds and cleaves dsRNA |
| PF00652.17 | Ricin_B_lectin | Domain | 24 | 124 | 124 | 26.3 | 5.3e-06 | CL0066 | Ricin-type beta-trefoil lectin domain | |
| PF00076.17 | RRM_1 | Domain | 1 | 52 | 70 | 24.9 | 1e-05 | CL0221 | RNA recognition motif. (a.k.a. RRM, RBD, or RNP domain) | ssRNA binding |
| PF00665.21 | rve | Domain | 22 | 119 | 120 | 41.9 | 8.4e-11 | CL0219 | Integrase core domain | Retroelement |
| PF00665.21 | rve | Domain | 37 | 119 | 120 | 32.3 | 7.7e-08 | CL0219 | Integrase core domain | Retroelement |
| PF00665.21 | rve | Domain | 22 | 119 | 120 | 42.6 | 5e-11 | CL0219 | Integrase core domain | Retroelement |
| PF00077.15 | RVP | Domain | 11 | 72 | 100 | 22.2 | 8.7e-05 | CL0129 | Retroviral aspartyl protease | Retroelement |
| PF00077.15 | RVP | Domain | 9 | 55 | 100 | 22.8 | 5.8e-05 | CL0129 | Retroviral aspartyl protease | Retroelement |
| PF00077.15 | RVP | Domain | 10 | 72 | 100 | 23.1 | 4.5e-05 | CL0129 | Retroviral aspartyl protease | Retroelement |
| PF03619.11 | Solute_trans_a | Family | 159 | 271 | 274 | 47.1 | 1.7e-12 | No_clan | Organic solute transporter Ostalpha | |
| PF00085.15 | Thioredoxin | Domain | 18 | 89 | 104 | 31.2 | 1.2e-07 | CL0172 | Thioredoxin |  |
| PF00240.18 | ubiquitin | Domain | 26 | 65 | 69 | 34.6 | 7.7e-09 | CL0072 | Ubiquitin family |  |
| PF00400.27 | WD40 | Repeat | 13 | 38 | 39 | 13.3 | 0.052 | CL0186 | WD domain, G-beta repeat | |
| PF00642.19 | zf-CCCH | Family | 8 | 26 | 27 | 25.0 | 9.7e-06 | No_clan | Zinc finger C-x8-C-x5-C-x3-H type (and similar) | DNA/RNA binding |
| PF00642.19 | zf-CCCH | Family | 8 | 26 | 27 | 25.3 | 7.9e-06 | No_clan | Zinc finger C-x8-C-x5-C-x3-H type (and similar) | DNA/RNA binding |
| PF00642.19 | zf-CCCH | Family | 8 | 26 | 27 | 24.6 | 1.3e-05 | No_clan | Zinc finger C-x8-C-x5-C-x3-H type (and similar) | DNA/RNA binding |
| PF00642.19 | zf-CCCH | Family | 6 | 26 | 27 | 20.7 | 0.00021 | No_clan | Zinc finger C-x8-C-x5-C-x3-H type (and similar) | DNA/RNA binding |
| PF00642.19 | zf-CCCH | Family | 6 | 26 | 27 | 21.3 | 0.00014 | No_clan | Zinc finger C-x8-C-x5-C-x3-H type (and similar) | DNA/RNA binding |
| PF00098.18 | zf-CCHC | Domain | 1 | 17 | 18 | 25.3 | 8.7e-06 | CL0511 | Zinc knuckle | DNA/RNA binding |
| PF00098.18 | zf-CCHC | Domain | 2 | 18 | 18 | 25.3 | 8.3e-06 | CL0511 | Zinc knuckle | DNA/RNA binding |
| PF00098.18 | zf-CCHC | Domain | 1 | 17 | 18 | 25.3 | 8.3e-06 | CL0511 | Zinc knuckle | DNA/RNA binding |
| PF00098.18 | zf-CCHC | Domain | 1 | 16 | 18 | 25.5 | 7.2e-06 | CL0511 | Zinc knuckle | DNA/RNA binding |
| PF00098.18 | zf-CCHC | Domain | 1 | 17 | 18 | 23.3 | 3.6e-05 | CL0511 | Zinc knuckle | DNA/RNA binding |
| PF00098.18 | zf-CCHC | Domain | 1 | 17 | 18 | 23.3 | 3.5e-05 | CL0511 | Zinc knuckle | DNA/RNA binding |
| PF00098.18 | zf-CCHC | Domain | 2 | 17 | 18 | 23.6 | 2.9e-05 | CL0511 | Zinc knuckle | DNA/RNA binding |
| PF00098.18 | zf-CCHC | Domain | 2 | 17 | 18 | 23.7 | 2.8e-05 | CL0511 | Zinc knuckle | DNA/RNA binding |
| PF00098.18 | zf-CCHC | Domain | 1 | 17 | 18 | 24.3 | 1.7e-05 | CL0511 | Zinc knuckle | DNA/RNA binding |
| PF00098.18 | zf-CCHC | Domain | 1 | 17 | 18 | 24.6 | 1.4e-05 | CL0511 | Zinc knuckle | DNA/RNA binding |
| PF00098.18 | zf-CCHC | Domain | 1 | 17 | 18 | 24.7 | 1.3e-05 | CL0511 | Zinc knuckle | DNA/RNA binding |
| PF00098.18 | zf-CCHC | Domain | 3 | 18 | 18 | 21.3 | 0.00016 | CL0511 | Zinc knuckle | DNA/RNA binding |
| PF00098.18 | zf-CCHC | Domain | 1 | 15 | 18 | 21.0 | 0.0002 | CL0511 | Zinc knuckle | DNA/RNA binding |

Additional file 1: Table S2- Primer sequences, melting temperature Tm (^o^C), and insert length (bp) for four *O. marina* genes.

| Gene | Forward Primer  (5’ to 3’) | Reverse Primer  (5’ to 3’) | Tm (^o^C) forward | Tm (^o^C) reverse | Insert length (bp) |
| --- | --- | --- | --- | --- | --- |
| TVP1 | TCT GCT CGG CGG TGT TTT GTT CG | ACG GGC CGC TGG TGT CCT TC | 62.3 | 65.1 | 238 |
| ACT | TCG TTC CCC ACC GTG ATG ATG TTC | TGG ACC TGG CTG GAC GAG ACT TGA | 61.1 | 63.7 | 225 |
| TUB | AGG GCT GCA GGG GTT CTT GAT GTT | CCT CGT TGT CCA GCT GGG TCG TTA | 63 | 62.7 | 232 |
| PR | GTT CCG TTC GTG TAC GTG GT | GAC AAA CGG GTA TGT GAG CC | 60.9 | 60.4 | 137 |

Additional file 1: Table S3 (next page): ***O. marina*** **encodes DNA repair and recombination proteins conserved in other eukaryotes.** Homologs of components of the machinery for base excision repair (BER), mismatch repair (MMR), nucleotide excision repair (NER), homologous recombination (HR), meiosis-specific homologous recombination (HR-M1), DNA polymerase subunits involved in repair (DNAP), editing and processing nucleases (EPN), post-replication repair (PRR), chromatin structure relevant to repair (CS), the DNA damage checkpoint (DDC), DNA replication licencing (DRL), and DNA damage response (DDR) are present in *O. marina.* Data identified in the complete genome sequences of humans (*H. sapiens*), yeast (*S. cerevisiae*), kinetoplastids (*T. brucei*), parabasalids (*T. vaginalis*), apicomplexans (*T. gondii, C. parvum,* and genome sequence survey of *A. taiwanensis*), and a dinoflagellate (*P. marinus*) is compared with the *O. marina* ESTs

| **BER** | parp1 | + |  | + | + | + |  |  | + |  |  |
| --- | --- | --- | --- | --- | --- | --- | --- | --- | --- | --- | --- |
|  | parp2 | + |  |  |  |  |  |  | + | **+** |  |
| **MMR** | MLH1 | + | + | + | +++ | + | + | + | + | **+** |  |
| **NER** | RPA1/ScRFA1 | + | + | + | + | + | + | + | + | **+** |  |
|  | ERCC2/XPD/ScRad3 | + | + | + | + | + | + | + | + | **+** |  |
| **DNAP** | polD1/ScCDC2/DNAPdelta | + | + |  | + | + | + | + | + | **+** |  |
|  | polE/ScPOL2/DNAP epsilon | + | + |  | + | + | + | + | + | **+** |  |
|  | PCNA/ScPOL30/Mus209 | + | + |  | + | + | + | + | + | **+** |  |
| **HR** | Mre11 | + | + | + | + | + | + | + | + | **+** |  |
|  | Rad51 | + | + | + | + | + | + | + |  | **+** |  |
|  | Brca1 | + |  |  |  |  |  |  |  | **+** |  |
|  | SMC3 | + | + | + | +++ | + | + | + | + | **+** |  |
|  | SMC2/hCAPE | + | + | + | + | + | + | + | + | **+** |  |
|  | SMC4/hCAPC | + | + | + | ++ | + | + | + | + | **+** |  |
| **HR-M1** | SPO11-1 | + | + | + | + |  | + |  |  |  |  |
|  | SPO11-2 | – | – | + | – | + | + |  |  | **+** |  |
|  | Hop2 | + | + | + | ++ | + | + |  | + | **+** |  |
|  | Mns1 | + |  | + | + | + |  |  | + | **+** |  |
| **EPN** | FEN1/DNaseIV | + |  | + | + | + | + | + | + | **+** |  |
| **RPRR** | UBE2A/Rad6A/ScRad6/UbcD6 | + | + |  | + | + | + | + | + | **+** |  |
|  | UBE2B/Rad6B/ | + |  |  |  | + | + | + | + | **+** |  |
| **CS** | BLM/SGS1 | + |  |  |  | + | + | + | + | **+** |  |
|  | WRN/RecQ5 | + |  | + | +++ |  | + | + | + | **+** |  |
| **DDC** | Rad17/ScRad24 | + | + |  | + | + | + |  | + | **+** |  |
|  | chek1/ScRad27/GRP/Chk1 | + | + |  |  | + | + | + | + | **+** |  |
|  | chek2/ScRad53/Chk2 | + | + |  |  | + | + | + | + | **+** |  |
| **DRL** | MCM3 | + | + | + | + | + | + | + | + | **+** |  |
|  | MCM5 | + | + | + | + | + | + |  | + | **+** |  |
|  | MCM7 | + | + | + | + | + | + |  | + | **+** |  |
| **DDR** | ScSUC1 |  | + |  | + | + | + |  | + | **+** |  |

Additional file 1: Table S4: The *O. marina* EST dataset contains a number of sequences with hits to proteins involved in transcriptional regulation and splicing. Listed below are reciprocal hits with a database built with curated proteins from *H. sapiens* and *S. cerevisiae*.

| **Category** | **gene** | **function** | **clusters** |
| --- | --- | --- | --- |
| mRNA splicing | Lsm2 | U6 associated Sm-like | 1 |
|  | Ist3 | component of U2 snRNP | 3 |
|  | Cwc2 | member of NTC Nineteen Complex | 1 |
|  | Cdc40 | also a pre-mRNA splicing factor | 5 |
|  | Lsm7 | U6 associated Sm-like | 1 |
|  | Lea1 | component of U2 snRNP | 1 |
|  | Lsm3 | U6 associated Sm-like | 1 |
|  | Snu13 | U3 snoRNP-associated protein, RNA-binding | 1 |
|  | Snu114 | GTPase component of U5 snRNP | 5 |
|  | Smx2 | Sm G, a core SM protein | 1 |
|  | Smd2 | Sm D2, a core SM protein | 1 |
|  | Prp46 | member of NTC | 16 |
|  | Sub2 | component of TREX complex for mRNA export also involved in splicing | 5 |
|  | Prp28 | ATP dependent RNA helicase | 4 |
|  | Hsh49 | U2 snRNP associated splicing factor | 1 |
|  | Prp43 | ATP dependent RNA helicase of DEAH-box family | 5 |
|  | Tpt1 | tRNA phosphotransferase | 1 |
|  |  |  |  |
| Transcription | Pdr3 | transcriptional activator of drug-resistance |  |
|  | Ctk1 | positive transcription elongation factor | 5 |
|  | Rpt1 | ATPase of the 19S regulatory particle of 26S proteosome | 4 |
|  | Kin28 | serine-threonine protein kinase, a CDK and TFIIK subunit | 5 |
|  | Rpt3 | ATPase of the 19S regulatory particle of 26S proteosome | 4 |
|  | Dst1 | general transcription elongation factor TFIIS, enables RNA polymerase | 1 |
|  | Rpt6 | ATPase of the 19S regulatory particle of 26S proteosome | 4 |
|  | Hog1 | mitogen-activated protein kinase | 5 |
|  | Mcm5 | DNA Replication licensing factor | 5 |
|  | Caf40 | Component of the CCR4-NOT transcriptional complex | 1 |
|  | Pop2 | RNAse of DEDD superfamily | 2 |
|  | Rpt5 | ATPase of the 19S regulatory particle of 26S proteosome | 4 |
|  | Rpt4 | ATPase of the 19S regulatory particle of 26S proteosome | 4 |
|  | Sks1 | Serine-threonine protein kinase, suppressor of Snf3 | 5 |
|  | Ssn3 | suppressor of RNA Pol B, aka CDK8 | 5 |
|  | Arp7 | component of Swi/Snf and RSC chromatin remodeling complex | 5 |
|  | Sgv1 | cyclin-dependent kinase that functions in transcription | 5 |
