# Supplementary material for: Analysis of EST data of the marine protist Oxyrrhis marina, an emerging model for alveolate biology and evolution
Source: BMC Genomics. 2014 Feb 11;15:122. doi: 10.1186/1471-2164-15-122 (PMC3942190; doi:10.1186/1471-2164-15-122)
Supplement: Additional file 2: Figure S1 — O. marina encodes orthologs of meiosis-specific recombination genes. Aligned amino acid sites were analyzed by PhyML with an invarying and 8 γ-distributed substitution rate categories and the LG substitution model. Numbers at the nodes indicate % bootstrap support (≥ 50%) from 1000 replicates. O. marina Spo11 is closely related to apicomplexan Spo11-2. 218 sites, LnL = –10981.3. [file 1471-2164-15-122-S2.doc]

Additional file 2: Figure S1: *O. marina* encodes orthologs of meiosis-specific recombination genes. Aligned amino acid sites were analyzed by PhyML with an invarying and 8 γ-distributed substitution rate categories and the LG substitution model. Numbers at the nodes indicate % bootstrap support (> 50%) from 1000 replicates. *O. marina* Spo11 is closely related to apicomplexan Spo11-2. 218 sites, LnL= –10981.3.
