# Supplementary material for: Analysis of EST data of the marine protist Oxyrrhis marina, an emerging model for alveolate biology and evolution
Source: BMC Genomics. 2014 Feb 11;15:122. doi: 10.1186/1471-2164-15-122 (PMC3942190; doi:10.1186/1471-2164-15-122)
Supplement: Additional file 3: Figure S2 — O. marina encodes orthologs of meiosis-specific recombination genes. Aligned amino acid sites were analyzed by PhyML with an invarying and 8 γ-distributed substitution rate categories and the LG substitution model. Numbers at the nodes indicate% bootstrap support (≥ 50%) from 1000 replicates. O. marina Hop2 is most closely related to its ortholog in Perkinsus marinus, within the alveolates. 172 sites, LnL = –6417.0. [file 1471-2164-15-122-S3.doc]

Additional file 3: Figure S2: *O. marina* encodes orthologs of meiosis-specific recombination genes. Aligned amino acid sites were analyzed by PhyML with an invarying and 8 γ-distributed substitution rate categories and the LG substitution model. Numbers at the nodes indicate % bootstrap support (> 50%) from 1000 replicates. *O. marina* Hop2 is most closely related to its ortholog in *Perkinsus marinus,* within the alveolates. 172 sites, LnL= –6417.0.
