# Supplementary material for: Analysis of EST data of the marine protist Oxyrrhis marina, an emerging model for alveolate biology and evolution
Source: BMC Genomics. 2014 Feb 11;15:122. doi: 10.1186/1471-2164-15-122 (PMC3942190; doi:10.1186/1471-2164-15-122)
Supplement: Additional file 4: Figure S3 — Phylogeny of representative L-lactate permease LctP proteins indicates that O. marina lctP is most closely related to lctP in diatoms and an icthyosporean (S. arctica), which are derived from a clade of marine bacterial lctP homologs. 502 amino acid sites were analyzed by PhyML with an invarying and 8 γ-distributed substitution rate categories and the LG substitution model. Numbers at the nodes indicate % support (≥ 50%) from 1000 bootstrap replicates. LnL = – 23076.2. No other eukaryotic homologs were identified by BLASTp searches of the JGI, Broad Institute, or NCBI non-redundant databases, nor by tBLASTn searches of dbEST-others, with an e-value cutoff of 1. Some of the highly similar Neisseria and Haemophilus orthologous protein sequences were excluded from the phylogeny shown here. [file 1471-2164-15-122-S4.doc]

Additional file 4: Figure S3: Phylogeny of representative L-lactate permease LctP proteins indicates that *O. marina* *lctP* is most closely related to *lctP* in diatoms and an icthyosporean (*S. arctica*)*,* which are derived from a clade of marine bacterial *lctP* homologs. 502 amino acid sites were analyzed by PhyML with an invarying and 8 γ-distributed substitution rate categories and the LG substitution model. Numbers at the nodes indicate % support (> 50%) from 1000 bootstrap replicates. LnL= – 23076.2. No other eukaryotic homologs were identified by BLASTp searches of the JGI, Broad Institute, or NCBI non-redundant databases, nor by tBLASTn searches of dbEST-others, with an e-value cutoff of 1. Some of the highly similar *Neisseria* and *Haemophilus* orthologous protein sequences were excluded from the phylogeny shown here.
